# Assimilated Rank in the Navy

**Published:** 1850-05

**Authors:** 


### ASSIMILATED RANK IN THE NAVY.

We most earnestly commend to our readers, and to the profession generally, the manly appeal of our correspondent, as set forth in his letter below. It tells its own story so eloquently as to need no urging on our part. The matters complained of have been so long and so patiently endured, that those in authority seem to have lost sight of the claims of the medical officers of the navy to be treated as gentlemen. The medical departments of both services have always had our warmest sympathies, and we cordially hope that a universal response will be made by the profession to this appeal in their behalf. We also append the memorial, which it is proposed to distribute to the profession throughout the country. To any who may fail to receive it, and may feel interested in seeing their medical brethren obtain their just demands, we suggest that copies of the memorial should be made, signatures obtained, and then transmitted to Philadelphia, as requested by our correspondent.

Dear Sir:—You are requested to examine the subject of "A Memorial in behalf of the Medical Officers of the Navy of the United States," and, if you approve of its object, to give it the support of your name, and induce others to join in an attempt to procure by law an assimilated rank for medical officers in the Navy corresponding to that legally established for the same class of officers in the Army of the United States.

Very soon after the close of the war with England,—when the value of medical services was strongly impressed upon the mind of officers of the navy generally—the medical officers asked to be assigned a definite rank. Their petition was sustained by the opinion of the Secretary of the Navy (B. W. Crowninshield), by the Board of Navy Commissioners, in official communications, dated January, 1817, and addressed to the Naval Committee of the Senate. In May, 1816, nine captains signed an address to the Secretary of the Navy, in which they say, "We consider the medical department of such great importance to the navy of our country, that no reasonable measures ought to be omitted which could have a tendency to retain in the service the professional ability of those gentlemen who, by their experience, knowledge, zeal and humanity, have procured the esteem and confidence of those with whom they have been associated; and we also beg leave to express our belief that no reasonable inducements would be objected to by Congress to procure for those who are engaged in a perilous service, and who are constantly exposed to the diseases of all climates, the best medical aid which the country affords. To effect this, it must be obvious that the rank and pecuniary emolument ought to bear some proportion to what gentlemen of professional eminence would be entitled in private life."—In December, 1816, four captains addressed the Secretary of the Navy on the same subject. They say: "We have heard with pleasure that it is the intention of the medical officers of the navy to address a respectful memorial to you, requesting that measures might be taken by the Department to procure for them a definite rank in the service, an increase of pay, and the establishment by law of the rank of hospital surgeon."

When these memorials were presented, there were but thirty captains in the navy; and if, to the thirteen signers of these addresses, the three Navy Commissioners who approved of their object be added, it is fair to infer that they represented the general opinion of the navy on the subject. The names of the captains who signed the address were Samuel Evans, Joseph Bainbridge, S. Angus, James Renshaw, Geo. W. Rogers, James T. Leonard, Edward Trenchard, James Jones, L. Warrington, William Bainbridge, Isaac Hull, D. Deacon, Alexander S. Wadsworth, and the Navy Commissioners were John Rodgers, Stephen Decatur, and David Porter.

In the year 1824, through the representations of several distinguished surgeons, the present system of examining candidates for the medical department, prior to admission into the navy, and of assistant surgeons previous to promotion, was begun, which has been very influential in securing for the naval service a competent medical corps. An improvement in the condition of medical officers began about the same period; but it was not until August, 1846, that the efforts to assign them a definite position in the military organization of which they are members were successful. At that time the Honorable Secretary of the Navy issued the following:—

" *General Order.*

" Surgeons of the fleet, and Surgeons of more than twelve years, will rank with Commanders;

" Surgeons of less than twelve years, with Lieutenants;

"Passed-assistant Surgeons, next after Lieutenants;
"Assistant Surgeons not passed, next after Masters.
"Commanding and Executive officers of whatever grade, when on duty, will take precedence over all medical officers.
"This order confers no authority to exercise military command, and no additional right to quarters.

"George Bancroft.

"Navy Department, August 31, 1846."

Under this General Order, a question of precedence arose in October, 1849, on a mixed board of officers, assembled at Washington, D. C., between a Commander, whose commission is dated in February, 1847, and a Surgeon, whose commission is dated in April, 1831, and who therefore ranks with commanders from Apr. 1843, when he was a surgeon "of more than twelve years." The question, which name should be first signed to the report of the Board, was referred to the Secretary of the Navy. But the commander demurred to the legal authority of the Department to issue or enforce the order. The Honorable Secretary of the Navy has not yet made known his decision on this point. The effect of his delay for so long a period, now six months, has been to provoke remonstrance and encourage demonstration against the authority of the Department on the part of those officers of the line, including some passed midshipmen, who think the question can be legally decided by Congress alone, and in this manner the whole subject has been again opened to discussion and agitation.

It is said one "commander-in-chief" assumes that all general orders become obsolete the moment the Secretary of the Navy, who may have issued them, retires from office; he virtually assumes that the authority of the office of Secretary of the Navy dies every four years, or at least whenever a new administration comes into power.

In the Buffalo "Morning Express," of March 20th, is the following—

"*Naval Circular.*

"It seems to be impossible for the commanding officer of a squadron to issue any order calculated to *control*, in the slightest degree, the *course* and *wishes* of a *Purser*, but he promptly produces some circular of the many, obsolete or *otherwise*, with which the whole class appears to be provided, and presents it as an order from the Navy Department, and intended to operate as a check upon his command.

"The present Honorable Secretary of the Navy has not furnished me with these particular circulars or instructions, but he has furnished me with laws for the better government of the navy.

"The regulations of the Navy Department, approved by the President of the United States (as far as they are in use), and my own regulations, for the method and government of the squadron, will be enforced on board the ship under your command.

"I am, very respectfully, your obedient servant,
(Signed) "Chas. W. Morgan.
"Com. U. S. Naval Forces, Mediterranean.

"P. S.—As it is very apparent that great confusion and insubordination in the squadron arise out of the constant presentation, by the Pursers, of circulars, orders, and instructions from the different branches of the Navy Department, not properly authorized as such upon their face, by the authority of the Secretary of the Navy for their issue,—I have to direct, that the commanders of the different vessels composing the naval force under my command, will allow *no order whatsoever*, not heretofore sanctioned by me, to be carried into effect, without *my authority* therefor, until I am further instructed by the Honorable Secretary of the Navy.

"Respectfully,

(Signed) "CHAS. W. MORGAN.

"Captain ———, Commanding U. S. Ship ———, Mediterranean."

For similar assumption in 1843 or 1844, this same gentleman was deprived of command in the Mediterranean. Judge UPSHUR, then Secretary of the Navy, wrote him substantially as follows:

"An officer who debates, instead of obeying the orders of the Department, is unfit to be trusted with the interests of the government. Your successor has, therefore, been appointed, and you will return to the United States in the way most convenient to yourself."

Whatever rests upon the authority, judgment, caprice, or facility of disposition of an individual, is ever uncertain and unstable. For this reason, the medical officers of the Navy desire that a law may be enacted to give them a definite rank, so that all those who are disposed to demur, to cavil, to quibble, or to question the authority of the Department, may no longer find cause to exercise their ingenuity.

To illustrate the necessity and propriety of a law to establish a definite rank for medical officers, as well as for others of the civil branch of the naval service, reference may be made to the "American State Papers," volume for naval affairs, in which is a record of an outrage committed by Captain Oliver H. Perry on John Heath, a captain of marines. Captain Perry, in his own cabin, "gave to Captain Heath a blow." This act, and its investigation by a naval court-martial, caused the commissioned officers of the Mediterranean squadron to address a memorial to the Senate of the United States, dated Port Mahon, January 20th, 1817, of which the following is the concluding paragraph:

"The undersigned have no guarantee for the safety of their persons, but the use of those arms which the laws of their country have placed in their hands, and that personal strength with which nature has blessed them. To these means they must resort, and on them in future depend, unless the honorable Senate, to whom they look with filial confidence as the guardians of their rights, will, by timely interference, save them from the disagreeable alternative of relinquishing a profession to which they are enthusiastically attached; or, becoming, in every instance, the defenders not only of their characters but of their persons. Placed at a distance from their country, and without the immediate influence of its civil laws, your memorialists rely with confidence on the decision of the high tribunal to which they now solemnly appeal. Your memorialists trust it will not engross too much valuable time of the Senate, to institute an examination into the proceedings of this court in these two instances. They beg leave also to state, that a case occurred at Naples, in August last, between Captain J. O. Creighton, and Midshipman Marston, of the Washington, the decision on which they consider as tending to destroy the conviction which every officer ought to feel, while in the execution of the duties of his office, that the strong arm of the law is extended over him, equally for his protection during good conduct, and for his punishment when he deviates from its rules. If your memorialists have erred in making this appeal, they hope it will be attributed rather to an exuberance than a deficiency of good feeling; and they trust they will ever be fouud ready to obey the call and support the cause of their country in any contest, however unpromising to themselves as individuals.

"And your memorialists, as in duty bound, will ever pray.

"*Thomas Ap Catesby Jones*, Lieut., Navy.
*W. B. Shubrick*, Lieutenant.
R. T. Auchmuty, Lieutenant Marine Corps.
Christopher Ford, Lieut., Marine Corps.
George Pearce, Lieutenant, Navy.
Beverly Kennon, Lieutenant, Marines.
*Samuel L. Breese*, Lieutenant, Navy.
Thomas Nichols, Sailing Master.
*Robert F. Stockton*, Lieutenant, Navy.
Francis B. White, Lieutenant, Marines.
Joseph L. Kuhn, Lieutenant, Marines.
W. H. Watson, Lieutenant, Navy.
Wm. H. Cocke, Lieutenant, Navy.
H. B. Breckenridge, Captain, Marines.
*B. Washington*, Surgeon.
George B. English, Lieutenant, Marines.
*James Armstrong*, Lieutenant, U. S. Navy.
George Beale, Purser.
*C. S. M'Cauley*, Lieutenant, U. S. Navy.
Hyde Ray, Surgeon.
Chas. T. Stallings, Lieutenant, Navy.
E. W. Turner, Purser.
Joseph Cassin, Lieutenant, Navy.
Gustavus W. Spooner, Lieutenant, Navy.
Robert S. Kearney, Surgeon.
William Hall, Captain, Marines.
*John Harris*, Lieutenant, Marines.
Henry Ollcott, Lieutenant, Marines.
N. Webster, Lieutenant, Navy.
*S. H. Stringham*, Lieutenant, Navy.
*W. K. Latimer*, Lieutenant, Navy.
L. Roupeaug, Lieutenant, Navy.
Alex. H. Montgomery, Acting Surgeon.

ROBERT FIELD, Lieutenant, Navy.
N. L. MONTGOMERY, Lieutenant, Navy.
M. D. NICHOLSON, Lieutenant, Navy.
W. LAUGHTON, Lieutenant, Navy.
JOHN CADLE, Acting Surgeon.
JOHN W. PEACO, Surgeon.
M. C. ATWOOD, Purser.
J. L. MORRIS, Lieutenant, Navy."

The names of those who are still in the Navy are in italics.

It does not appear that the complainants obtained any substantial reply to their prayer.

Medical men employed in either branch of the military service of the country, are small detachments from the great body of the medical community; although laboring alone, they do not abandon a claim to the common interests and sympathies of the profession; and while upon the field, or on the ocean, they may regard themselves as its delegates or representatives, bound to submit to its common rules of ethics and policy. Until within a few years, there were no facilities for invoking the protection of their professional brothers; but now the profession is organized to some extent, in County, and in State societies, and the American Medical Association of the United States, it is hoped medical men included in military organizations will not appeal in vain.

Members of the medical profession serving in the army and navy are all alumni of some alma mater; and the professional parent may properly protect and encourage her offspring whenever protection and encouragement are needed.

Say then to alma mater;—do not permit your alumni to be forced below their proper position in any community of Americans, civil or military. You have prepared them in your halls to be physicians, and have recorded your declaration that they are worthy of confidence and respect. You have taught them their obligations; you may properly aid them in securing an observance of their rights, and a just consideration. Your alumni are refused a place relatively to those with whom they are associated; and are denied those conventional signs of respect which are peculiar to military communities, while they are subject in all things to martial laws and usages. In an exacting military organization they are without those rights which are given legally to one class, and are left to a contingent courtesy for the treatment they may receive.

In a similar spirit, call upon our fellow-alumni, and ask them individually, and in societies, wherever they may be, to examine the subject, and urge their friends and representatives in the National Legislature to extend over us the protection of law.

With this object, you are requested to date the memorial and address one copy to the Senate, or to one of the United States' Senators of your State, and another to the representative of the Congressional district in which you reside. After all the gentlemen who are willing to countenance this effort have signed, please return the papers to Philadelphia, addressed to Mr. F. Brown, Northeast corner of Fifth and Chestnut St., and endorsed "Medical Memorial." When all sections of the country are heard from, the whole of the papers will be forwarded to Washington at the same date.

It is sincerely hoped no unnecessary delay will be indulged in executing the request which is thus earnestly and respectfully preferred.

Very respectfully, &c.,

*A memorial in behalf of the Medical Officers of the Navy of the United States.*

The undersigned respectfully represent, that the medical officers of the Navy of the United States, until within a few years, have been without an assigned position relatively to others in the military community of which they are members. It being made manifest that the want of a relative position caused them to suffer, in many instances, grievous inconvenience and mortification, the Honorable the Secretary of the Navy issued a General Order, dated August 31st, 1846, for the purpose of assigning a relative position to medical officers in the Navy. This General Order was acceptable to the medical corps; it called forth an expression of gratification from the body of the medical profession of the Union, represented in the "American Medical Association," (May 1848,) because, in the language of the resolution, it "regards with pride and satisfaction the services rendered, and the position maintained by that portion of their profession associated with the Military Department of the country; and in consideration of the severe and arduous duties which the medical officers have performed, the risks and dangers to which they have been exposed in the performance of those duties, during a period of warfare, and in an unhealthy climate, it is deemed just and proper, by this Association, that their services should receive from the government, an acknowledgement corresponding to that awarded their brother officers." The members of the medical corps would be content with the assimilated rank thus conferred, although below that which has been justly given by law to their professional brothers in the Army of the United States. But as some officers of the line in the Navy have avoided obeying the General Order of August 31st, 1846, with impunity, and even question the authority of the Secretary of the Navy to issue the said order, which, in effect, confers no power on medical officers, and takes away neither power nor dignity from officers of the line; and as experience has shown, that observance of the order cannot interfere with the general discipline of the naval service, nor lessen its efficiency, your memorialists pray Congress will enact, that *the grades of medical officers of the Navy shall have the same degree of rank relatively to officers of the line in the Navy, as corresponding grades of medical officers in the Army now possess according to law, relatively to officers of the line in the Army*, provided *that the assimilated rank hereby conferred shall not entitle any medical officer in the Navy to increased pay, or to take precedence of any officer who may be in legal command of any post, station or vessel, to which said medical officer may be officially attached for duty.*

The medical officers of the Navy seek, in this measure, only what is just and reasonable; they do not ask a right to command officers of the line, nor any power not designated in their present commissions, nor exemption from any duty.

It is believed that members of the medical profession generally, throughout the United States, cannot be indifferent to any executive or legislative action, which may stamp a sign of low appreciation on the science of Medicine and Surgery when exercised in the military services of the nation. It is hoped, therefore, that the justice and propriety of their cause will not be less apparent, because it is urged against numbers by a comparatively small corps of men, separated only while on duty from the great body of their medical brothers, to which they are united by the common bonds of professional, social, and scientific fellowship. And, as in duty bound, your memorialists will ever pray.

